# Mitochondrial DNA variation and intervertebral disc degeneration: a genotypic analysis in a South African cohort

**DOI:** 10.1007/s11033-025-10394-6

**Published:** 2025-03-07

**Authors:** Megan Collins, Brendon Pearce

**Affiliations:** Genetics Department, Faculty of Agriscience, Stellenbosch University, Van Der Bijl Street, Stellenbosch, 7600 South Africa

**Keywords:** Intervertebral disc degeneration, mtDNA, Mitochondrial dysfunction

## Abstract

**Background:**

Non-communicable diseases are multifactorial in that they can be caused by genetic factors, age, sex and poor lifestyle choices. They are estimated to account for 71% of deaths globally with 80% of these deaths occurring in low- and middle-income countries. This is particularly true for Intervertebral Disc Degeneration associated with mitochondrial dysfunction. Interestingly, mitochondrial dysfunction can arise from mutations in both the nuclear and the mitochondrial genomes. The present study, therefore, aimed to determine if there is an association between mitochondrial DNA mutations associated with mitochondrial dysfunction and disc degeneration in a South African cohort, and in addition, generate genetic data for understudied mutations in African populations.

**Methods and results:**

Mutations were selected using a systematic literature review. DNA was collected using buccal swabs and extracted using a standard salt-lysis protocol. Mass-array genotyping was done for previously reported as well as novel mutations. GenAlEx (version 6.5), RStudio and SHEsis were used for statistical analyses. Although no significant associations were found, the identified polymorphic mutations C16223T, A10398G and A8536G were found to have higher mutant allele frequencies in case individuals indicating that had a larger cohort been used, significance may have been observed.

**Conclusions:**

This study was able to generate genotypic information for a South African cohort for both reported and understudied mutations. Furthermore, the identification of higher mutant allele frequencies for C16223T, A10398G and A8536G highlights the importance of considering these mutations in future studies using a larger cohort.

**Supplementary Information:**

The online version contains supplementary material available at 10.1007/s11033-025-10394-6.

## Introduction

Non-communicable diseases (NCDs) refer to pathological conditions that are multifactorial in that they can be caused by genetic factors, age, sex and poor lifestyle choices [1]. NCDs are estimated to account for 71% of deaths globally with 80% of these deaths occurring in low- and middle-income countries (LMICs) [2]. Current implementations to combat NCDs include prevention, management or reversal of the above-mentioned lifestyle factors that are modifiable. Furthermore, LMICs face further challenges to combat NCDs due to underfunding of healthcare systems and lack of proper equipment to accommodate patients with NCDs. Treating NCDs is also expensive and takes a long time. Health care systems, further prioritize infectious diseases such as HIV, malaria and maternal/child health over NCDs. Since NCDs are also understudied in these regions it makes it more difficult to create effective policies that address the financial burdens of NCDs in these households [2]. This is particularly true for Intervertebral Disc Degeneration (IVDD). IVDD is characterised by an imbalance between extracellular matrix (ECM) synthesis and degradation as well as increased apoptosis and senescence of nucleus pulposus cells (NPCs). The speed of IVDD progression is linked to high levels of ROS production as disc components are vulnerable to oxidative damage [3–5]. Studies have identified mutations exclusively in nuclear genes associated with this condition. These studies have specifically examined mutations in collagen and aggrecan genes, given their role as structural components of the intervertebral discs [6]. Interestingly, these studies have also elucidated that mitochondrial dysfunction can arise from mutations in both the nuclear genome and the mitochondrial genome [7].

Genome-wide association studies have provided valuable information regarding the heritable nature of complex diseases. The majority of this information, however, has been conducted in European (Caucasian) populations [8]. This limits the generalizability of these medications across diverse population groups. African populations are thought to harbour the most genetic diversity globally so much so that it is expected that most genotype–phenotype associations could potentially be found in these populations than anywhere else [9]. The inclusion of African populations in genetic studies is of important consideration since individuals in these populations have some of the lowest life expectancies globally and receive the lowest access to quality medical care. Populations in South Africa, specifically, within the same geographic regions have complex patterns of admixture and population structure [9]. However, disease variants within South African populations and their subsequent haplogroups are understudied and, whilst the haplogroups may be well characterised, significant disease variants found in other populations are unable to be generalized to South African populations as a result of the complexity of their genetic architecture. The aim of this study was, thus, to determine the genotypes of case and control individuals from a South African study cohort for disc degeneration to determine if there is an association between mitochondrial DNA (mtDNA) mutations associated with mitochondrial dysfunction and intervertebral disc degeneration. It further aims to not only generate genotypic data for underrepresented genetic groups but also generate genetic data for understudied mutations for which not much genetic data is available.

## Materials and methods

### Ethical clearance

Ethical clearance for this study was obtained from the Health Research Ethics Committee of Stellenbosch University (reference number: N23/04/034).

### Patient inclusion and exclusion criteria

Patients were included as cases in this study if they had a diagnosis of intervertebral disc degeneration (IVDD) confirmed by a clinician. Control individuals were included in this study if they did not have a pre-existing diagnosis of disc degeneration. Written and informed consent was given by all participants. Individuals were considered if they were between the ages of 18 and 80 and if they belonged to a South African population group and had any of the following comorbidities: Diabetes, hypertension, hyperlipidemia, arthritis and history of family arthritis. A total of 92 case individuals and 123 control individuals were included in the study for genotyping, however, due to limited amounts of available biological sample, only 60 cases and 102 controls produced usable genotypic data that could be used for downstream analysis.

### SNV selection

Mutations were obtained utilizing a systematic literature review and selected for analysis if they were associated with general mitochondrial dysfunction. From the list, mutations were modelled to hypothesize if they resulted in structural changes and subsequent dysfunction from which a genotyping assay was developed.

### DNA extractions

DNA was collected using buccal swabs and subsequently extracted using a standard salt-lysis protocol. Buccal swabs provide a non-invasive DNA collection method yielding sufficient quality and quantity of DNA [10]. The concentration (ng/$\upmu$ l) of DNA samples as well as quality (absorbance ratios) was assessed using a NanoDrop Spectrophotometer (ThermoFisher Scientific) and subsequently frozen at -20 °C until the sample was needed for genotyping.

### Primer design

Primers for PCR amplifications, SNP genotyping, and NGS sequencing were designed using the Primer3 software as well as Agena’s in-house design software. Allele-specific primers, indicated in Supplementary Table 1 were designed and then analyzed in OligoAnalyser and Eurofins.

### Mass array genotyping

The Agena MassArray System was used to genotype previously reported and novel SNVs discovered in this study. It is a primer single-base extension-based method that enables multiplexing up to 40 SNVs. This method is based on the molecular weight of each amplified fragment to detect a single base change or polymorphism.

### Allele-specific genotyping

Allele-specific genotyping was done manually for mutations that were not included in the mass array due to a lack of an Rs number (Identifier) or overlaps in product band sizes for SNVs too close together. These mutations were A8344G, T5578C, A8536G, T3394C and G3460A. PCR reactions were run using a Qiagen PCR kit (QIAGEN GmbH, D-40724 Hilden) according to the manufacturer’s instructions. PCR products were subsequently visualized via gel electrophoresis where the genotypes for each individual were called based on the observable difference in band sizes for each allele at each locus.

### Statistical analysis

Statistical analysis was performed using GenAlEx (version 6.5) [11] and RStudio (version 2024.04.2) (RStudio Team [12]. GenAlEx (version 6.5) [11] was used to generate data for Hardy–Weinberg Equilibrium, allele frequencies, the Shannon index, fixation indices and G-statistics. RStudio (version 2024.04.2) (RStudio Team [12] was used to model each mutation using logistic regression. Models included mutations along with age, ethnicity and sex as variables and diabetes, hypertension, hyperlipidemia, arthritis and history of family arthritis as comorbidities.

## Results

Below are the results showing demographic information for the study cohort used, a list of mutations that were selected for the genotyping assay and the data that was generated in GenAlEx (version 6.5) [11].

### Study cohort

#### Patients, demographics, and comorbidities

The study cohort consisted of 60 cases and 102 controls of which, where demographic data was available, 40 individuals were Cape Admixed, 84 were Caucasian, 2 were Zulu, 3 were Asian/Indian, 3 were Black and 3 were Indian. Of the case individuals 39 were females and 21 were males while for the controls 42 were females and 35 were males (Table 1). The co-morbidities of the study participants, as captured from the available medical records, are presented in Table 2.

**Table 1:** Ages of case and control individuals for study cohort

Age	Cases	Controls
18–19	0	8
20–29	1	47
30–39	3	2
40–49	12	3
50–59	16	5
60–69	12	11
70–79	12	1
80–89	3	0

**Table 2:** Comorbidities for case and control data

Comorbidity	Yes/No	Total	Cases	Controls
Smoker	Yes	30	14	16
	No	76	34	42
Diabetes	Yes	10	8	2
	No	110	41	69
Hypertension	Yes	30	23	7
	No	89	26	63
Hyperlipidemia	Yes	19	17	2
	No	100	32	68
Osteoarthritis	Yes	23	22	1
	No	69	27	42
Family history of osteoarthritis	Yes	28	20	8
	No	28	22	6

Mutations associated with general mitochondrial dysfunction that were selected for the genotyping assay for assessment of disc degeneration are presented in Table 3.

**Table 3:** Table showing mutations selected and included for downstream analysis

	Mutation	Rs ID	Locus	Heteroplasmy	Reference
1	A3243G	Rs199474657	MT-TER, MT-TL1	Yes	[13–18]
2	C3275T	Rs1057516057	MT-TL1	No	[19, 20]
3	A3302G	Rs1603218878	MT-TL1	Yes	[21], [20]
4	T3394C	Rs41460449	MT-ND1	Both	[22]
5	G3460A	Rs199476118	MT-ND1	Both	[23]
6	T3472C	Rs1603218973	MT-ND1	No	[24]
7	A4917G	Rs28357980	MT-ND2	Both	[25]
8	C5178A	Rs28357984	MT-ND2	No	[26]
9	T5578C		MT-TW	Yes	[27]
10	A8344G	Rs118192098	MT-TK	Yes	[28, 29]
11	A8536G	No RS	MT-ATP8, MT-ATP6	–	[30]
12	A10398G	Rs2853826	MT-ND3	Yes	[31]
13	G11778A	Rs199476112	MT-ND4	No	[32]
14	A12308G	Rs2853498	MT-TL2	Both	[33]
15	A14692G	Rs879192165	MT-TE	No	[34]
16	C16223T	Rs2853513	MT-HV1, MT-ATT, MT-CR, 7SDNA	–	[35]

### Hardy–weinberg equilibrium data

The Hardy–Weinberg equilibrium analysis (Table 4) was conducted using GenAlEx (version 6.5) [11].

**Table 4:** Table showing Hardy–Weinberg Equilibrium data for case and control individuals

Case/Control	Locus	DF	ChiSq	Prob	Signif
Case	C3275T	Monomorphic			
Case	A3302G	Monomorphic			
Case	T3472C	Monomorphic			
Case	A3243G	Monomorphic			
Case	G11778A	Monomorphic			
Case	A4917G	1	57.000	0.000	***
Case	C5178A	Monomorphic			
Case	A12308G	1	46.723	0.000	***
Case	C16223T	1	43.022	0.000	***
Case	A10398G	1	57.000	0.000	***
Case	A14692G	Monomorphic			
Case	A8344G	1	53.000	0.000	***
Case	T5578C	1	55.000	0.000	***
Case	A8536G	1	51.000	0.000	***
Case	T3394C	1	55.000	0.000	***
Case	G3460A	1	54.000	0.000	***
Control	C3275T	Monomorphic			
Control	A3302G	Monomorphic			
Control	T3472C	Monomorphic			
Control	A3243G	Monomorphic			
Control	G11778A	Monomorphic			
Control	A4917G	1	55.000	0.000	***
Control	C5178A	Monomorphic			
Control	A12308G	1	43.000	0.000	***
Control	C16223T	1	33.349	0.000	***
Control	A10398G	1	50.000	0.000	***
Control	A14692G	Monomorphic			
Control	A8344G	1	52.000	0.000	***
Control	T5578C	1	60.000	0.000	***
Control	A8536G	1	42.000	0.000	***
Control	T3394C	1	69.000	0.000	***
Control	G3460A	1	71.000	0.000	***

*(p-value < 0.05), **(p-value < 0.01), ***(p-value < *0.001), p* < *0.05 was considered significant*

From the genotyped mutations in the South African cohort, the following SNVs showed genetic variation: A4917G, A12308G, C16223T, A10398G, A8344G, T5578C, A8536G, T3394C and G3460A.

### Allele frequency data

The minor allele frequencies for the case and control populations were generated in GenAlEx (version 6.5) [11] along with minor allele frequencies from Ensembl for European, African, East Asian, American mixed and South Asian Population groups (Table 5).

**Table 5:** Minor allele frequency data generated for case and control populations along with minor allele frequency data for various population groups from Ensembl

Locus	Allele	Cases	Controls	EUR	AFR	EAS	AMR	SAS
C3275T	T	0	0	0,001	0.003	0		
A3302G	G	0	0	–	–	–	–	–
T3472C	C	0	0	–	–	–	–	–
A3243G	G	0	0	–	–	–	–	–
G11778A	A	0	0	0.001	0	0	–	–
A4917G	G	0,123	0,145	0,102	0,026	0,002	0,076	0,011
C5178A	A	0	0	0,002	0,005	0,179	–	–
A12308G	G	0,157	0,140	0,234	0,142	0,004	0,000	–
C16223T	T	0,436	0,227	–	–	–	–	–
A10398G	G	0,544	0,380	0,151	0,613	0,610	0,375	0,000
A14692G	G	0	0	–	–	–	–	–
A8344G	G	0,019	0,058	–	–	–	–	–
T5578C	C	0,091	0,133	–	–	–	–	–
A8536G	G	0,451	0,310	–	–	–	–	–
T3394C	C	0,036	0,043	0,012	0,001	0,031	0,000	0,000
G3460A	A	0,111	0,141	–	–	–	–	–

*Minor allele frequencies for the sample population compared to representative global populations, abbreviated as follows: Cases: Individuals with disc degeneration diagnosis; Controls: Individuals without disc degeneration; EUR: European population; AFR: African population; EAS: East Asian Population; AMR: Latin American populations; SAS: South Asian Populations; YRI: Yoruba; and ‘- ‘: No population data.*

### Fixation indices

Below, in Table 6, are the fixation indices, generated using GenAlEx (version 6.5) [11] for both the case and control populations which shows where certain mutations were monomorphic and where there was variation present. When considering the fixation indices for the genotyped mutations, fixation indices at a value of 1 show that the population is monomorphic for the wild-type allele, whereas, if the fixation index deviated from 1 it showed that variation was present for that specific mutation.

**Table 6:** The fixation indices for various mutations for case individuals

Population	Locus	N	Na	Ne	I	Ho	He	uHe	F
	C3275T	54	1.000	1.000	0.000	0.000	0.000	0.000	#N/A
	A3302G	56	1.000	1.000	0.000	0.000	0.000	0.000	#N/A
	T3472C	58	1.000	1.000	0.000	0.000	0.000	0.000	#N/A
	A3243G	55	1.000	1.000	0.000	0.000	0.000	0.000	#N/A
	G11778A	55	1.000	1.000	0.000	0.000	0.000	0.000	#N/A
	A4917G	57	2.000	1.275	0.372	0.000	0.215	0.217	1.000
	C5178A	56	1.000	1.000	0.000	0.000	0.000	0.000	#N/A
	A12308G	54	2.000	1.361	0.435	0.019	0.265	0.268	0.930
	C16223T	47	2.000	1.968	0.685	0.021	0.492	0.497	0.957
	A10398G	57	2.000	1.985	0.689	0.000	0.496	0.501	1.000
	A14692G	57	1.000	1.000	0.000	0.000	0.000	0.000	#N/A
	A8344G	53	2.000	1.038	0.094	0.000	0.037	0.037	1.000
	T5578C	55	2.000	1.198	0.305	0.000	0.165	0.167	1.000
	A8536G	51	2.000	1.981	0.688	0.000	0.495	0.500	1.000
	T3394C	55	2.000	1.075	0.156	0.000	0.070	0.071	1.000
	G3460A	54	2.000	1.246	0.349	0.000	0.198	0.199	1.000
	C3275T	37	1.000	1.000	0.000	0.000	0.000	0.000	#N/A
	A3302G	37	1.000	1.000	0.000	0.000	0.000	0.000	#N/A
	T3472C	54	1.000	1.000	0.000	0.000	0.000	0.000	#N/A
	A3243G	37	1.000	1.000	0.000	0.000	0.000	0.000	#N/A
	G11778A	52	1.000	1.000	0.000	0.000	0.000	0.000	#N/A
	A4917G	55	2.000	1.331	0.415	0.000	0.249	0.251	1.000
	C5178A	54	1.000	1.000	0.000	0.000	0.000	0.000	#N/A
	A12308G	43	2.000	1.316	0.404	0.000	0.240	0.243	1.000
	C16223T	44	2.000	1.541	0.536	0.045	0.351	0.355	0.871
	A10398G	50	2.000	1.891	0.664	0.000	0.471	0.476	1.000
	A14692G	50	1.000	1.000	0.000	0.000	0.000	0.000	#N/A
	A8344G	52	2.000	1.122	0.221	0.000	0.109	0.110	1.000
	T5578C	60	2.000	1.301	0.393	0.000	0.231	0.233	1.000
	A8536G	42	2.000	1.747	0.619	0.000	0.427	0.433	1.000
	T3394C	69	2.000	1.091	0.179	0.000	0.083	0.084	1.000
	G3460A	71	2.000	1.319	0.406	0.000	0.242	0.244	1.000

*N sample size at specific locus, Na number of observed alleles at specific locus, Ne measure of allele diversity, I Shannon’s index to measure genetic diversity, Ho proportion of heterozygotes observed in the population, He the proportion of heterozygotes expected under Hardy–Weinberg equilibrium, uHe corrected measure of He that accounts for sample size bias, F fixation index measuring deviation from Hardy–Weinberg equilibrium at a locus.*

## Discussion

### Sample population, IVDD and demographics trends

#### IVDD, age and sex

IVDD, as previously stated, is an age-related disorder due to ageing being responsible for increases in oxidative stress. Due to intervertebral disc (IVD) cells being vulnerable to oxidative stress, the effects of ageing result in loss of IVD viability and function and cells will start to release matrix proteases and chemokines which degrade cells further [36]. Even though these variables were not significant in the logistic regression analysis, it is apparent that the majority of the cases were above the age of 50 (43 individuals) with very few individuals being in the younger age categories (16 individuals) (Table 1) which is consistent with literature that disc degeneration is age-related and is more likely to affect individuals over the age of 50 [37]. It is thus plausible that the lack of association between IVDD and age observed in this study is merely due to the small sample size and due to convenience sampling, there was a mismatch between the age categories in the control group which would have been required for an accurate assessment of the effects of ageing with this disorder.

Roberts et al. (2021) found that disc degeneration is more prevalent in young males than in young females. A potential hypothesis for this could be due to increased mechanical stress and physical injuries experienced by men. Women, however, in the later years of their lives have experienced increased severity over males which is most likely explained by menopausal changes resulting in changes in bone density. Furthermore, they found that beyond the 5th decade of life, it appeared that sex no longer had a significant role in disease manifestation highlighting that this disorder is potentially more associated with age than it is with sex [38]. In the cohort for the present study, no significant association for sex and disc degeneration was observed, however, the case individuals consisted of more women (39 women) than men (21 men). A larger study cohort would need to be considered to determine if the findings identified by *Roberts *et al. (2021) hold true for a larger South African cohort.

#### IVDD and ethnicity

The study cohort consisted of individuals that are part of the Cape Admixed, Caucasian, Asian, Zulu, African and Indian population groups, with the majority of the samples coming from Cape Admixed (40 individuals) and Caucasian populations (84 individuals). The skewed distribution of ethnicities is explained by their collection from pain clinics in Panorama and Cape Town City Bowl, primarily housing Cape Admixed and Caucasian individuals. South Africa is known for its substantial population diversity with 79% of the population being broadly classified as African, 2.5% Asian and 9.6% European. Cape Admixed individuals comprise approximately 9% of South Africa’s total population and 54% of the Western Cape’s population [39]. The Caucasian individuals in the Western Cape, specifically the Afrikaaner individuals, have ancestry predominantly with European populations (95.3%) comprising mainly Dutch, German and French with non-European estimates being between 5.5–7.2% [40].

The lack of heterozygotes observed (Table 4) is likely due to the Wahlund effect which describes a reduction in heterozygosity due to the subdivision of a population into smaller, genetically distinct subpopulations. Since Cape Admixed individuals can be divided into subgroups with varying proportions of African, European and Asian ancestry [39] it is possible that when considering these subgroups together, the genetic variation within this population group may not adhere to the expected Hardy–Weinberg Equilibrium trends. It is, further, possible that the nature of mtDNA inheritance may exacerbate the Wahlund effect due to mtDNA not undergoing recombination with changes in linkage only being due to mutational hotspots that exist within the genome [41]. No significant associations were found regarding disease phenotype, mutations and ethnicity. This could be due to the small sample size and the lack of sufficient numbers of individuals within each ethnic group. Future studies should have a larger cohort with more individuals from different population groups so that associations regarding ethnicity will be more accurate.

### Sample population, IVDD, and comorbidity trends

The comorbidities identified in this study include diabetes, hypertension, hyperlipidemia, and arthritis and will be discussed further in detail.

#### IVDD and Type-2 diabetes mellitus (T2D)

Among the comorbidities, diabetes stands out due to its well-documented impact on disc degeneration. *Liu X *et al*.* (2018) demonstrated that patients with T2D are prone to more severe intervertebral disc degeneration compared to non-diabetic individuals. The mechanism is linked to hyperglycemia, which triggers the overproduction of advanced glycation end products (AGEs). These AGEs accumulate in the disc tissue, accelerating cellular damage and promoting degeneration [42].

Interestingly, despite this known pathophysiology, only a small number of participants in this study were diagnosed with diabetes. This low prevalence raises the possibility that, within this specific cohort, diabetes may not have played a significant role in driving disc degeneration. This could suggest that other risk factors may be more dominant contributors in this population. Further investigation could help clarify the nuances of diabetes’ role in disc pathology across different groups.

#### IVDD, hypertension and hyperlipidemia

Hypertension, a secondary comorbidity in this study, plays a critical role in disc degeneration through its contribution to systemic inflammation [43]. Elevated blood pressure triggers the release of chemokines, which recruit immune cells that, once activated, amplify the inflammatory cascade within the disc tissue [44]. The prevalence of hypertension among cases surpasses that of controls, aligning with its potential role in disc degeneration. It is plausible that with a larger cohort, a statistically significant association between hypertension and disc degeneration might have been revealed.

Similarly, hyperlipidemia was assessed as a contributing factor to disc degeneration. Elevated lipid levels and the dysregulation of proinflammatory adipokines associated with lipid metabolism have been linked to disc cell senescence and apoptosis [45]. Most individuals with hyperlipidemia were among the cases, consistent with existing literature. This finding suggests that a larger sample size may have unveiled a stronger, significant association between hyperlipidemia and disc degeneration, reinforcing its potential influence on the pathology of IVDD.

#### IVDD and osteoarthritis

Osteoarthritis was the final comorbidity examined in this study. This degenerative joint disease is characterized by the breakdown of articular cartilage, accompanied by changes in the subchondral bone, meniscus, tendons, ligaments, muscles, and synovial inflammation [46]. What makes osteoarthritis particularly relevant to this analysis is the substantial overlap in the molecular pathways and degenerative processes shared with intervertebral disc degeneration (IVDD), further supporting its inclusion as a key comorbidity (Fine et al., 2023).

Our data revealed a higher prevalence of osteoarthritis among cases compared to controls, aligning with existing literature (Fine et al., 2023) that highlights the biological connection between these disorders. Although this trend was not statistically significant in our sample, the findings suggest that a larger cohort could have revealed a more pronounced association, strengthening the argument for the interplay between osteoarthritis and disc degeneration.

### Population genetic interplay between IVDD and mitochondrial dysfunction

#### Monomorphic sites

From the genotyped data, the following mutations were identified as monomorphic in both cases and controls: C3275T, A3302G, T3472C, A3243G, G11778A, C5178A, and A14692G (Tables 4 and 5). According to MitoMap and existing literature, A3302G and A3243G are heteroplasmic mutations [47, 48].

*Van Den Bosch *et al*.* (2004) demonstrated that the A3302G mutation, affecting tRNA^Leu^, significantly reduces complex I activity in the mitochondrial respiratory chain and leads to abnormal mitochondrial RNA-processing [47]. Similarly, the A3243G mutation, also present in tRNA^Leu^, has been implicated in complex I. The severity and presentation of clinical outcomes, however, are complex, with variability observed between individuals and organ systems due to differing heteroplasmy thresholds [48].

Interestingly, no allele frequency data for these two mutations are available in the South African cohort (Table 5). Despite their known associations with disease in heteroplasmic states, the absence of variation suggests that these mutations, while significant in other populations, may not play a role in the disease phenotypes observed in South Africans.

The remaining monomorphic mutations (C3275T, T3472C, G11778A, C5178A, and A14692G) are identified as homoplasmic according to MitoMap and literature [20, 24, 49–51] *Ding *et al*.* (2018) found that the C3275T mutation causes significant thermodynamic changes in tRNA^Leu^, potentially altering its secondary structure. T3472C, G11778A and C5178A have been shown to decrease complex I activity resulting in subsequent mitochondrial dysfunction [24, 32, 52]. Lastly, the A14692G mutation in tRNA^Glu^ disrupts its conformation and stability resulting in mitochondrial dysfunction [53].

These findings raise intriguing questions about the population-specific impact of mtDNA mutations. While these variants are linked to various disease phenotypes in global populations, their monomorphic status in the South African cohort suggests they may not contribute to disease manifestation or progression here. This observation could be influenced by the distinct haplogroups these populations belong to.

The divergence in haplogroups could be a key factor. Specific mtDNA mutations may occur more frequently in certain haplogroups, and younger populations may still harbour mutations that were purged in older populations due to selective pressure. African populations, being among the oldest, may have eliminated more harmful mutations over time, while these mutations persist in younger population groups today [54]. This hypothesis underscores the importance of considering evolutionary history and genetic diversity when studying disease-associated mutations across different ethnic groups.

### Allele frequencies, heteroplasmy and population variance in variant sites

#### Polymorphic sites

The genotyping analysis of the study cohort revealed that mutations A4917G, A12308G, C16223T, A10398G, A8344G, T5578C, A8536G, T3394C, and G3460A displayed variation in the genotyping data. Literature and databases like MitoMap indicate that certain mutations are heteroplasmic, such as A10398G, A8344G, and T5578C [27, 31, 55]. The heteroplasmic A10398G mutation, located in the MT-ND3 gene, is particularly significant due to its association with altered mitochondrial respiratory chain Complex I structure when the mutation load is high [31]. The A8344G mutation, found in the tRNA^Lys^ gene, impairs protein synthesis, leading to the inhibition of oxidative phosphorylation. This mutation, however, is rare [55], therefore, its absence from population allele frequency data in Table 5 is consistent with this fact. The T5578C mutation, occurring in the tRNA^Trp^ gene, has been shown to alter tRNA structure and cause subsequent mitochondrial dysfunction (Y. Liu et al., 2016).

Other mutations, including A4917G [56, 57], A12308G [58], T3394C [59], and G3460A [60, 61] have been reported in both heteroplasmic and homoplasmic states. The A4917G mutation in MT-ND2, when present homoplasmically, has been associated with an increased risk of macular degeneration due to mitochondrial dysfunction in a study conducted on Caucasian populations [62]. The A12308G mutation in tRNA^Leu^, homoplasmic in an Iranian cohort, has been linked to cancer development [58] and is also associated with increased disease risk in European populations, particularly among those in haplogroup U [63]. The T3394C mutation, located in MT-ND1, is reported to be associated with metabolic diseases and deafness in a Chinese population [59]. The G3460A mutation in MT-ND1 affects the structure and function of ND1, reducing Complex I activity and contributing to Leber’s hereditary optic neuropathy (LHON) [61].

The interplay between heteroplasmic and homoplasmic states across populations reveals that whether a mutation manifests in one state or the other may be population-specific, necessitating tailored investigations across different demographic groups. Mutations A8536G and C16223T have been associated with disease phenotypes, although the A8536G mutation remains understudied and lacks clarity on whether it presents as heteroplasmic or homoplasmic. The C16223T mutation, despite being studied, has not been definitively classified as either heteroplasmic or homoplasmic due to its location in the mitochondrial D-loop [35].

The genotyping variation observed in Table 5, showing that both wild-type and mutant alleles appear in both cases and controls, suggests the possibility of heteroplasmic states within South African populations. Notably, the highest mutant allele frequencies were observed for C16223T, A10398G, and A8536G (Table 5). For C16223T, the mutant allele was more frequent in cases than controls, although it was still present in both groups, suggesting the possibility of a heteroplasmic threshold. The same pattern was observed for A10398G, which may also indicate a heteroplasmic threshold. Although this mutation has been associated with protective effects against certain diseases [64], the allele frequencies observed in this South African cohort suggest that it may contribute to disease phenotypes in this population due to the mutant allele having a higher prevalence in the case individuals. Allele frequency data for A8536G also points to a potential heteroplasmic threshold. Like C16223T, A8536G is understudied and lacks sufficient evidence regarding its heteroplasmic or homoplasmic nature, yet the data from this cohort suggest a similar trend.

Finally, the variations in heteroplasmic and homoplasmic states observed in this study highlight the complexity and population-specific nature of mitochondrial mutations.

### Fixation indices, genetic differentiation statistics and shannon indices

When considering the fixation indices (Table 6) it is observed that even though A10398G and A8536G had allele frequencies that indicated a potential heteroplasmic threshold, a fixation index of 1 is observed for both these mutations. C16223T, however has a value of F = 0,959 for the case individuals and a value of F = 0,860 for the controls. The fixation index for the case individuals shows that the population is not completely monomorphic for the wild type allele. The control fixation index shows a strong fixation for the wild type allele; however, it is lower than that of the case fixation index which shows that the mutant allele is less common in the controls.

From the genetic differentiation data (Supplementary Table S2), C16223T shows a significant positive value of 0.037. This shows that C16223T may have meaningful genetic differentiation between the case and control populations which suggests that its allele frequencies differ significantly and subsequently contribute to the disease phenotype. This highlights that this mutation may be a potential candidate for further studies regarding its role in disease susceptibility.

When analyzing genetic diversity within and among populations in the case and control individuals for polymorphic mutations (Supplementary Table S3), it is observed that C16223T, A10398G and A8536G had significant population differentiation. When accounting for permutations (Supplementary Table S4), A10398G and A8536G are no longer significant, however, C16223T remains significant which indicates that the variation observed may not be due to stochastic changes.

### Logistic regression analysis

For each of the above logistic regression analysis models (Supplementary Table S5), no significant variables were observed, however, due to the identification of monomorphic and polymorphic data within the study cohort and interesting observations regarding mutant allele frequencies (Table 5), it can be hypothesized that the lack of significance may be due to the small sample size of this study. Furthermore, a cohort consisting of more individuals from each ethnic group should be considered for greater genetic representation along with matching of variables for age and sex amongst cases and controls. Furthermore, demographic data such as ethnicity in most cases is self-reported and thus is not always accurate. For a more accurate model these concerns would need to be taken into consideration for future studies wishing to continue this line of research.

### Implications for diagnosis and prediction

Disc degeneration is a leading cause of lower back pain [65], and increasing evidence links mitochondrial dysfunction to this degenerative process [66]. This pilot study, while exploratory, holds significant potential for uncovering the genetic mechanisms underlying lower back pain (LBP) driven by disc degeneration. Given the limited treatment options currently available for disc degeneration—primarily conservative therapies or invasive spinal surgeries, both of which often result in further complications [67]—the study’s findings offer a new avenue for research into genetic factors, even though no significant associations were detected in this instance.

Focusing specifically on a South African population, the study uncovered intriguing insights into potential mutations linked to disc degeneration due to mitochondrial dysfunction. Notably, the A10398G, C16223T and A8536G mutations exhibited higher mutant allele frequencies, suggesting that with a larger cohort, potential associations could have emerged. These findings hint at the possibility that such mitochondrial mutations may play a role in the progression of disc degeneration.

If future studies can identify these mutations—or others—as reliable diagnostic markers, it could lead to earlier intervention strategies. Early detection of these genetic risk factors may open the door to novel therapeutic interventions, ultimately improving patient outcomes and reducing the need for invasive surgeries. Although the study did not yield significant associations, its implications lay the groundwork for further exploration into the genetic underpinnings of disc degeneration, particularly within diverse populations like South Africa.

### Inferences for South African populations

NCDs are estimated to soon be the leading cause of disability and death in South African populations [68]. Given that lower back pain (LBP) itself is an NCD and among the leading causes of disability globally, with its effects being detrimental to the workforce of populations [69], it is imperative that potential disease variants be identified to better understand this complex disorder on a molecular level. Current LBP treatments include conservative therapy and then spinal surgery if conservative methods are unsuccessful [67]. This calls for increased research regarding personalized medicine and biological insights into population-specific variants for these NCDs. However, even though the need for this has been identified, satisfactory contributions to this area are lacking due to increased investment in more urgent medical developments in infectious diseases and maternal/child healthcare [2]. South Africa, furthermore, has a shortage of skilled personnel to interpret genomic data as well as a shortage of infrastructure and technology to support the research that needs to occur for the advancement of personalized medicine [70].

In this pilot study, an attempt was made to find an association between mtDNA variations associated with mitochondrial dysfunction and individuals with disc degeneration. Although no significant associations were found, variation was observed among some mutations and potentially, had the study cohort been larger, a significant association may have been observed for A8536G, A10398G and C16223T. Furthermore, this study emphasizes that significance cannot simply be generalized across populations and that population-specific studies need to be conducted in South Africa to tailor both the diagnostic and treatment pathways for NCDs along with furthering our understanding of LBP due to disc degeneration.

## Future research

To better understand this disease in South African populations, future research must involve a significantly larger study cohort, as the current cohort size was limited. Notably, the presence of certain mutations suggests that a more extensive investigation is warranted. Variants such as C16223T, A10398G and A8536G, which demonstrated higher mutant allele frequencies in this study, should be prioritized in larger-scale studies to assess their prevalence in South African population groups, which may have been underrepresented due to sample size constraints. These studies could further consider principal component analysis (PCA) which would allow for accurate control over genetic differences between different population groups and to determine if any potentially significant disease variants are ancestral. Furthermore, studies could assess hetero/homoplasmy of variants that were identified as polymorphic within the South African cohort. Future studies should, furthermore, have a greater representation of both sexes, various age groups and the comorbidities that were selected for this study to accurately assess the potential molecular mechanisms contributing to the manifestation of this complex disorder.

## Limitations

Limitations of this study include the small cohort. The small sample size doesn’t allow for a well-rounded representation of the diverse population groups within South Africa with only a few individuals being genotyped per ethnic group. It is also possible that due to the small sample size bias may have occurred in the study and skewed the results. Furthermore, bias may also have occurred due to the nature in which demographic data was both obtained and recorded as ethnicity was self-reported and the persistent substantial divide in clinical data capture standards between clinical sites.

## Conclusion

From this pilot study, mtDNA mutations associated with mitochondrial dysfunction could be assessed for their potential association with disc degeneration using a South African cohort. Firstly, monomorphic mutations could be differentiated from mutations that had variation within this study cohort and from this, mutations with higher allele frequencies for mutant alleles could be identified namely A8536G, A10398G and C16223T. Although no significant associations were identified, this information will help to aid future studies regarding disc degeneration in a South African cohort in terms of choosing which mutations to assess further. Since the identified mutations form part of overlapping diseases, this data could also be used and applied to a South African cohort for assessment of the other disease phenotypes that these mutations have been associated with.

## Supplementary Information

Below is the link to the electronic supplementary material.

Supplementary file1 (DOCX 56 KB)

## Data Availability

The datasets generated during and/or analysed during the current study are available from the corresponding author upon reasonable request.
