# Supplementary material for: Mitochondrial DNA variation and intervertebral disc degeneration: a genotypic analysis in a South African cohort
Source: Mol Biol Rep. 2025 Mar 7;52(1):288. doi: 10.1007/s11033-025-10394-6 (PMC11889028; doi:10.1007/s11033-025-10394-6)
Supplement: Supplementary file 1 — Supplementary file1 (DOCX 56 KB) [file 11033_2025_10394_MOESM1_ESM.docx]

**Supplementary Data**

***Supplementary Table S1:*** *Primers used for allele-specific genotyping*

| **SNP** | **Primer Type** | **Primer** | **Amplicon Size (bp)** | **Tm (eurofins)** |
| --- | --- | --- | --- | --- |
| **A8344G** | WT reverse | CATTTCACTGTAAAGAGGTGTTGGT | 553 | 59,7 |
|  | M reverse | TTGGGGCATTTCACTGTAAAGAGGTGTTGGC | 563 | 69,5 |
|  | Comm forward | CCTCCCATCCCTACGCATCCTTTA |  | 64,4 |
| **T5578C** | WT reverse | TTGCAGTCCTTAGCTGTTACA | 492 | 55,9 |
|  | M reverse | GAGTGGGGTTTTGCAGTCCTTAGCTGTTACG | 502 | 69,5 |
|  | Comm forward | CTACCGCATTCCTACTACTCAACT |  | 61 |
| **A8536G** | WT forward | CCTGAGAACCAAAATGAACGAAA | 644 | 57,1 |
|  | M forward | TATAACAAACCCTGAGAACCAAAATGAACGAAG | 654 | 64,5 |
|  | Comm reverse | CTTGGATTAAGGCGACAGCGATTT |  | 61 |
| **T3394C** | WT forward | ACCGAACGAAAAATTCTAGGCT | 464 | 56,5 |
|  | M forward | CCTAATGCTTACCGAACGAAAAATTCTAGGCC | 474 | 66,9 |
|  | Comm reverse | GGCAGGAGTAATCAGAGGTGTT |  | 60,3 |
| **G3460A** | WT reverse | CTTTGGTGAAGAGTTTTATGGC | 403 | 56,5 |
|  | M reverse | TTTAGGGGCTCTTTGGTGAAGAGTTTTATGGT | 413 | 65,6 |
|  | Comm forward | GGAGTAATCCAGGTCGGTTTCTAT |  | 61 |

**4.4.5 Genetic Differentiation Statistics**

The genetic differentiation statistics are presented in **Supplementary Table S2** and were calculated using GenAlEx (version 6.5)(Peakall & Smouse, 2012).

***Table S2:*** *Genetic differentiation statistics for mitochondrial DNA loci*

| Locus | Gst | G'stN | G'stH | G''st | Dest | P (Gst) |
| --- | --- | --- | --- | --- | --- | --- |
| C3275T | - | - | - | - | - | - |
| A3302G | - | - | - | - | - | - |
| T3472C | - | - | - | - | - | - |
| A3243G | - | - | - | - | - | - |
| G11778A | - | - | - | - | - | - |
| A4917G | -0.008 | -0.016 | -0.013 | -0.021 | -0.005 | 0.682 |
| C5178A | - | - | - | - | - | - |
| A12308G | -0.010 | -0.020 | -0.016 | -0.026 | -0.007 | 0.733 |
| C16223T | 0.039 | 0.074 | 0.097 | 0.130 | 0.061 | 0.037 |
| A10398G | 0.018 | 0.034 | 0.052 | 0.068 | 0.035 | 0.100 |
| A14692G | - | - | - | - | - | - |
| A8344G | 0.001 | 0.001 | 0.001 | 0.001 | 0.000 | 0.286 |
| T5578C | -0.004 | -0.009 | -0.006 | -0.011 | -0.002 | 0.471 |
| A8536G | 0.010 | 0.020 | 0.029 | 0.038 | 0.018 | 0.179 |
| T3394C | -0.008 | -0.016 | -0.009 | -0.017 | -0.001 | 0.663 |
| G3460A | -0.006 | -0.012 | -0.010 | -0.016 | -0.004 | 0.633 |

*Where “-“ indicates no value as the SNV is monomorphic*

***Supplementary Table S3:*** *Population Shannon index statistics without permutations*

| **Pop 1** | **Pop 2** | **Ct1** | **Ct2** | **Wt1** | **Wt2** | **sHa1** | **sHa2** | **sHu** | **sHua** | **10^sHa1** | **10^sHa2** | **10^sHu** | **10^sHua** | **Nm** | **G** | **DF** | **Chi Prob** | **Chi Signf** |
| --- | --- | --- | --- | --- | --- | --- | --- | --- | --- | --- | --- | --- | --- | --- | --- | --- | --- | --- |
| **C3275T** |  |  |  |  |  |  |  |  |  |  |  |  |  |  |  |  |  |  |
| Pop 1 | Pop 2 | 108 | 74 | 0.593 | 0.407 | 0.000 | 0.000 | 0.000 | 0.000 | 1.000 | 1.000 | 1.000 | 1.000 | #DIV/0! | 0.000 | 0 | 1.000 | ns |
| **A3302G** |  |  |  |  |  |  |  |  |  |  |  |  |  |  |  |  |  |  |
| Pop 1 | Pop 2 | 112 | 74 | 0.602 | 0.398 | 0.000 | 0.000 | 0.000 | 0.000 | 1.000 | 1.000 | 1.000 | 1.000 | #DIV/0! | 0.000 | 0 | 1.000 | ns |
| **T3472C** |  |  |  |  |  |  |  |  |  |  |  |  |  |  |  |  |  |  |
| Pop 1 | Pop 2 | 116 | 108 | 0.518 | 0.482 | 0.000 | 0.000 | 0.000 | 0.000 | 1.000 | 1.000 | 1.000 | 1.000 | #DIV/0! | 0.000 | 0 | 1.000 | ns |
| **A3243G** |  |  |  |  |  |  |  |  |  |  |  |  |  |  |  |  |  |  |
| Pop 1 | Pop 2 | 110 | 74 | 0.598 | 0.402 | 0.000 | 0.000 | 0.000 | 0.000 | 1.000 | 1.000 | 1.000 | 1.000 | #DIV/0! | 0.000 | 0 | 1.000 | ns |
| **G11778A** |  |  |  |  |  |  |  |  |  |  |  |  |  |  |  |  |  |  |
| Pop 1 | Pop 2 | 110 | 104 | 0.514 | 0.486 | 0.000 | 0.000 | 0.000 | 0.000 | 1.000 | 1.000 | 1.000 | 1.000 | #DIV/0! | 0.000 | 0 | 1.000 | ns |
| **A4917G** |  |  |  |  |  |  |  |  |  |  |  |  |  |  |  |  |  |  |
| Pop 1 | Pop 2 | 114 | 110 | 0.509 | 0.491 | 0.162 | 0.180 | 0.171 | 0.000 | 1.451 | 1.514 | 1.483 | 1.001 | #### | 0.248 | 1 | 0.619 | ns |
| **C5178A** |  |  |  |  |  |  |  |  |  |  |  |  |  |  |  |  |  |  |
| Pop 1 | Pop 2 | 112 | 108 | 0.509 | 0.491 | 0.000 | 0.000 | 0.000 | 0.000 | 1.000 | 1.000 | 1.000 | 1.000 | #DIV/0! | 0.000 | 0 | 1.000 | ns |
| **A12308G** |  |  |  |  |  |  |  |  |  |  |  |  |  |  |  |  |  |  |
| Pop 1 | Pop 2 | 108 | 86 | 0.557 | 0.443 | 0.189 | 0.176 | 0.183 | 0.000 | 1.545 | 1.498 | 1.525 | 1.000 | #### | 0.121 | 1 | 0.728 | ns |
| **C16223T** |  |  |  |  |  |  |  |  |  |  |  |  |  |  |  |  |  |  |
| Pop 1 | Pop 2 | 94 | 88 | 0.516 | 0.484 | 0.297 | 0.233 | 0.277 | 0.011 | 1.984 | 1.709 | 1.892 | 1.025 | 18.931 | 9.046 | 1 | 0.003 | ** |
| **C10398G** |  |  |  |  |  |  |  |  |  |  |  |  |  |  |  |  |  |  |
| Pop 1 | Pop 2 | 114 | 100 | 0.533 | 0.467 | 0.299 | 0.288 | 0.300 | 0.006 | 1.992 | 1.943 | 1.996 | 1.014 | 64.145 | 5.778 | 1 | 0.016 | * |
| **A14692G** |  |  |  |  |  |  |  |  |  |  |  |  |  |  |  |  |  |  |
| Pop 1 | Pop 2 | 114 | 100 | 0.533 | 0.467 | 0.000 | 0.000 | 0.000 | 0.000 | 1.000 | 1.000 | 1.000 | 1.000 | #DIV/0! | 0.000 | 0 | 1.000 | ns |
| **A8344G** |  |  |  |  |  |  |  |  |  |  |  |  |  |  |  |  |  |  |
| Pop 1 | Pop 2 | 106 | 104 | 0.505 | 0.495 | 0.041 | 0.096 | 0.070 | 0.002 | 1.098 | 1.247 | 1.176 | 1.005 | #### | 2.252 | 1 | 0.133 | ns |
| **T5578C** |  |  |  |  |  |  |  |  |  |  |  |  |  |  |  |  |  |  |
| Pop 1 | Pop 2 | 110 | 120 | 0.478 | 0.522 | 0.132 | 0.171 | 0.153 | 0.001 | 1.356 | 1.481 | 1.423 | 1.002 | #### | 1.041 | 1 | 0.308 | ns |
| **A8536G** |  |  |  |  |  |  |  |  |  |  |  |  |  |  |  |  |  |  |
| Pop 1 | Pop 2 | 102 | 84 | 0.548 | 0.452 | 0.299 | 0.269 | 0.290 | 0.005 | 1.990 | 1.857 | 1.949 | 1.011 | #### | 3.920 | 1 | 0.048 | * |
| **T3394C** |  |  |  |  |  |  |  |  |  |  |  |  |  |  |  |  |  |  |
| Pop 1 | Pop 2 | 110 | 138 | 0.444 | 0.556 | 0.068 | 0.078 | 0.073 | 0.000 | 1.169 | 1.196 | 1.184 | 1.000 | #DIV/0! | 0.000 | 1 | 1 | ns |
| **G3460A** |  |  |  |  |  |  |  |  |  |  |  |  |  |  |  |  |  |  |
| Pop 1 | Pop 2 | 108 | 142 | 0.432 | 0.568 | 0.151 | 0.177 | 0.166 | 0.000 | 1.417 | 1.502 | 1.466 | 1.001 | #### | 0.491 | 1 | 0.483 | ns |
| **Mean over loci** |  |  |  |  |  |  |  |  |  |  |  |  |  |  |  |  |  |  |
| Pop 1 | Pop 2 | #### | #### | 0.524 | 0.476 | 0.102 | 0.104 | 0.105 | 0.002 | 1.266 | 1.271 | 1.274 | 1.004 | #### |  |  |  |  |

***Pop 1*** *– cases,* ***Pop 2*** *– controls,* ***Ct1*** *– Sum of case individuals,* ***Ct2*** *– Sum of control individuals,* ***Wt1*** *– Frequencies for cases,* ***Wt2*** *– Frequencies for controls,* ***sHa1*** *– Shannon diversity index for cases,* ***sHa2*** *– Shannon index diversity,* ***sHu*** *– Unbiased Shannon diversity index,* ***sHua*** *– Average unbiased Shannon diversity index,* ***10^sHa1*** *Effective number of alleles based on Shannon index for cases,* ***10^sHa2*** *– Effective number of alleles based on Shannon index for controls,* ***10^sHu*** *– Effective number of alleles based on unbiased Shannon index,* ***10^sHua*** *– Effective number of alleles based on average unbiased Shannon index,* ***Nm*** *– Gene flow estimated from diversity data,* ***G*** *– Genetic differentiation between populations,* ***DF*** *– Degrees of freedom for test of differentiation,* ***Chi Prob*** *– Probability value from chi-square test,* ***Chi Signf*** *– Significance of chi-square test (significant if p < 0.05)*

***Supplementary Table S4:*** *Population Shannon index statistics with permutations*

| **Pop 1** | **Pop 2** | **Ct1** | **Ct2** | **Wt1** | **Wt2** | **sH(WP1)** | **sH(WP2)** | **sH(WP)** | **sH(GT)** | **sH(AP)** | **2^sH(WP)** | **2^sH(GT)** | **2^sH(AP)** | **DivWt** | **D’** | **O’=1-D’** | **P(rand >=data)** |
| --- | --- | --- | --- | --- | --- | --- | --- | --- | --- | --- | --- | --- | --- | --- | --- | --- | --- |
| **C3275T** |  |  |  |  |  |  |  |  |  |  |  |  |  |  |  |  |  |
| Pop 1 | Pop 2 | 108 | 74 | 0.593 | 0.407 | 0.000 | 0.000 | 0.000 | 0.000 | 0.000 | 1.000 | 1.000 | 1.000 | 1.965 | 0.000 | 1.000 | 1.000 |
| **A3302G** |  |  |  |  |  |  |  |  |  |  |  |  |  |  |  |  |  |
| Pop 1 | Pop 2 | 112 | 74 | 0.602 | 0.398 | 0.000 | 0.000 | 0.000 | 0.000 | 0.000 | 1.000 | 1.000 | 1.000 | 1.958 | 0.000 | 1.000 | 1.000 |
| **T3472C** |  |  |  |  |  |  |  |  |  |  |  |  |  |  |  |  |  |
| Pop 1 | Pop 2 | 116 | 108 | 0.518 | 0.482 | 0.000 | 0.000 | 0.000 | 0.000 | 0.000 | 1.000 | 1.000 | 1.000 | 1.999 | 0.000 | 1.000 | 1.000 |
| **A3243G** |  |  |  |  |  |  |  |  |  |  |  |  |  |  |  |  |  |
| Pop 1 | Pop 2 | 110 | 74 | 0.598 | 0.402 | 0.000 | 0.000 | 0.000 | 0.000 | 0.000 | 1.000 | 1.000 | 1.000 | 1.962 | 0.000 | 1.000 | 1.000 |
| **G11778A** |  |  |  |  |  |  |  |  |  |  |  |  |  |  |  |  |  |
| Pop 1 | Pop 2 | 110 | 104 | 0.514 | 0.486 | 0.000 | 0.000 | 0.000 | 0.000 | 0.000 | 1.000 | 1.000 | 1.000 | 1.999 | 0.000 | 1.000 | 1.000 |
| **A4917G** |  |  |  |  |  |  |  |  |  |  |  |  |  |  |  |  |  |
| Pop 1 | Pop 2 | 114 | 110 | 0.509 | 0.491 | 0.162 | 0.180 | 0.171 | 0.171 | 0.000 | 1.482 | 1.483 | 1.001 | 2.000 | 0.001 | 0.999 | 0.731 |
| **C5178A** |  |  |  |  |  |  |  |  |  |  |  |  |  |  |  |  |  |
| Pop 1 | Pop 2 | 112 | 108 | 0.509 | 0.491 | 0.000 | 0.000 | 0.000 | 0.000 | 0.000 | 1.000 | 1.000 | 1.000 | 2.000 | 0.000 | 1.000 | 1.000 |
| **A12308G** |  |  |  |  |  |  |  |  |  |  |  |  |  |  |  |  |  |
| Pop 1 | Pop 2 | 108 | 86 | 0.557 | 0.443 | 0.189 | 0.176 | 0.183 | 0.183 | 0.000 | 1.524 | 1.525 | 1.000 | 1.987 | 0.001 | 0.999 | 0.792 |
| **C16223T** |  |  |  |  |  |  |  |  |  |  |  |  |  |  |  |  |  |
| Pop 1 | Pop 2 | 94 | 88 | 0.516 | 0.484 | 0.297 | 0.233 | 0.266 | 0.277 | 0.011 | 1.846 | 1.892 | 1.025 | 1.999 | 0.049 | 0.951 | 0.025 |
| **A10398G** |  |  |  |  |  |  |  |  |  |  |  |  |  |  |  |  |  |
| Pop 1 | Pop 2 | 114 | 100 | 0.533 | 0.467 | 0.299 | 0.288 | 0.294 | 0.300 | 0.006 | 1.969 | 1.996 | 1.014 | 1.996 | 0.027 | 0.973 | 0.093 |
| **A14692G** |  |  |  |  |  |  |  |  |  |  |  |  |  |  |  |  |  |
| Pop 1 | Pop 2 | 114 | 100 | 0.533 | 0.467 | 0.000 | 0.000 | 0.000 | 0.000 | 0.000 | 1.000 | 1.000 | 1.000 | 1.996 | 0.000 | 1.000 | 1.000 |
| **A8344G** |  |  |  |  |  |  |  |  |  |  |  |  |  |  |  |  |  |
| Pop 1 | Pop 2 | 106 | 104 | 0.505 | 0.495 | 0.041 | 0.096 | 0.068 | 0.070 | 0.002 | 1.169 | 1.176 | 1.005 | 2.000 | 0.011 | 0.989 | 0.292 |
| **T5578C** |  |  |  |  |  |  |  |  |  |  |  |  |  |  |  |  |  |
| Pop 1 | Pop 2 | 110 | 120 | 0.478 | 0.522 | 0.132 | 0.171 | 0.152 | 0.153 | 0.001 | 1.420 | 1.423 | 1.002 | 1.998 | 0.005 | 0.995 | 0.468 |
| **A8536G** |  |  |  |  |  |  |  |  |  |  |  |  |  |  |  |  |  |
| Pop 1 | Pop 2 | 102 | 84 | 0.548 | 0.452 | 0.299 | 0.269 | 0.285 | 0.290 | 0.005 | 1.929 | 1.949 | 1.011 | 1.991 | 0.021 | 0.979 | 1.169 |
| **T3394C** |  |  |  |  |  |  |  |  |  |  |  |  |  |  |  |  |  |
| Pop 1 | Pop 2 | 110 | 138 | 0.444 | 0.556 | 0.068 | 0.078 | 0.073 | 0.073 | 0.000 | 1.184 | 1.184 | 1.000 | 1.987 | 0.000 | 1.000 | 1.000 |
| **G3460A** |  |  |  |  |  |  |  |  |  |  |  |  |  |  |  |  |  |
| Pop 1 | Pop 2 | 108 | 142 | 0.432 | 0.568 | 0.151 | 0.177 | 0.166 | 0.166 | 0.000 | 1.465 | 1.466 | 1.001 | 1.982 | 0.002 | 0.998 | 0.625 |
| **Mean over loci** |  |  |  |  |  |  |  |  |  |  |  |  |  |  |  |  |  |
| Pop 1 | Pop 2 | 109.250 | 100.875 | 0.524 | 0.467 | 0.102 | 0.104 | 0.104 | 0.105 | 0.002 | 1.270 | 1.274 | 1.004 | 1.998 | 0.007 | 0.993 | 0.221 |

***Pop 1*** *– cases,* ***Pop 2*** *– controls,* ***Ct1*** *– Sum of case individuals,* ***Ct2*** *– Sum of control individuals,* ***Wt1*** *– Frequencies for cases,* ***Wt2*** *– Frequencies for controls,* ***sH(WP1)*** *– Shannon diversity index for within population diversity for cases,* ***sH(WP2)*** *– Shannon diversity index for within-population diversity for controls,* ***sH(WP)*** *– Average Shannon diversity index across all within-population comparisons,* ***sH(GT)*** *– Shannon diversity index for total genetic diversity across all populations,* ***sH(AP)*** *Shannon diversity index for among-population genetic diversity,* ***2^sH(WP)*** *Effective number of alleles with populations,* ***2^sH(GT)*** *Effective number of alleles for total diversity across populations,* ***2^sH(AP)*** *Effective number of alleles for among-population diversity,* ***DivWt*** *– Weighted diversity across populations,* ***D’*** *– proportion of total diversity that is due to among-population differences,* ***O’ = 1 - D’*** *– Measure of similarity,* ***P(rand >=data)*** *– Probability that observed differentiation is due to random chance*

Represented above are the Shannon indices with and without permutations. As observed in **Table B.2,** mutations C16223T, A10398G and A8536G are significant, however, after permutations (**Table B.3**), only C16223T remains significant.

***Supplementary Table S5:*** *Logistic regression analysis of case and control individuals including each mutation and relevant variables and comorbidities*

|  | **Estimate** | **Standard error** | **Z value** | **Pr(>\|z\|)** |
| --- | --- | --- | --- | --- |
| Intercept (Status) | 2.713e+01 | 7.733e+05 | 0 | 1 |
| A4917G (1) | -4.663e+01 | 6.344e+05 | 0 | 1 |
| A12308G (1) | 1.381e+01 | 8.278e+05 | 0 | 1 |
| C16223T (1) | 1.291e+01 | 5.037e+05 | 0 | 1 |
| A10398G (1) | -1.322e+01 | 4.191e+05 | 0 | 1 |
| A8344G (1) | -6.765e+01 | 1.075e+06 | 0 | 1 |
| T5578C (1) | 3.553e-02 | 7.558e+05 | 0 | 1 |
| A8536G (1) | -4.499e+00 | 3.482e+05 | 0 | 1 |
| G3460A (1) | 1.704e+01 | 4.205e+05 | 0 | 1 |
| Smoker (1) | -2.114e+01 | 1.923e+05 | 0 | 1 |
| Diabetes (1) | -1.096e+01 | 2.354e+05 | 0 | 1 |
| Hypertension (1) | -1.786e+01 | 3.010e+05 | 0 | 1 |
| Hyperlipidemia (1) | -2.203e+00 | 2.851e+05 | 0 | 1 |
| Osteoarthritis (1) | -4.340e+00 | 3.399e+05 | 0 | 1 |
| Family history of osteoarthritis (1) | -6.941e+00 | 3.223e+05 | 0 | 1 |
| Ethnicity (Cape Admixed) | 4.137e+01 | 5.950e+05 | 0 | 1 |
| Ethnicity (Caucasian) | 3.281e+01 | 5.902e+05 | 0 | 1 |
| Ethnicity (Zulu) | NA | NA | NA | NA |
| Sex (Male) | -9.978e+00 | 3.680e+05 | 0 | 1 |
| Age (50-59) | -1.383e+01 | 2.843e+05 | 0 | 1 |
| Age (60-69) | 6.984e+00 | 4.074e+05 | 0 | 1 |
| Age (70-79) | -3.812e+00 | 4.881e+05 | 0 | 1 |
| Age (80-89) | -1.482e+00 | 5.929e+05 | 0 | 1 |

*“1” – presence of mutant allele or comorbidity*

Represented above is the logistic regression analysis for each mutation that was included in the study for downstream analysis along with each of the relevant variables and comorbidities. T3394C was excluded from the model due to very minimal differences between cases and controls and thus being treated as having one level in the model instead of two levels consisting of individuals with and without the mutation.
